# Design of Tailor-Made Biopolymer-Based Capsules for Biological Application by Combining Porous Particles and Polysaccharide Assembly

**DOI:** 10.3390/pharmaceutics15061718

**Published:** 2023-06-13

**Authors:** Cléa Chesneau, Laura Larue, Sabrina Belbekhouche

**Affiliations:** Université Paris Est Creteil, CNRS, Institut Chimie et Matériaux Paris Est, UMR 7182, 2 Rue Henri Dunant, 94320 Thiais, France

**Keywords:** polysaccharide, porous particles, polyelectrolyte capsules, drug delivery systems, biological application

## Abstract

Various approaches have been described in the literature to demonstrate the possibility of designing biopolymer particles with well-defined characteristics, such as size, chemical composition or mechanical properties. From a biological point of view, the properties of particle have been related to their biodistribution and bioavailability. Among the reported core–shell nanoparticles, biopolymer-based capsules can be used as a versatile platform for drug delivery purposes. Among the known biopolymers, the present review focuses on polysaccharide-based capsules. We only report on biopolyelectrolyte capsules fabricated by combining porous particles as a template and using the layer-by-layer technique. The review focuses on the major steps of the capsule design, i.e., the fabrication and subsequent use of the sacrificial porous template, multilayer coating with polysaccharides, the removal of the porous template to obtain the capsules, capsule characterisation and the application of capsules in the biomedical field. In the last part, selected examples are presented to evidence the major benefits of using polysaccharide-based capsules for biological purposes.

## 1. Introduction

The progress and promise of drug delivery systems (DDS) opens new routes to tackle therapy problems in the treatment of several diseases, mainly because free drugs may diffuse non-specifically [1,2]. Fundamental research covering different scientific and technological areas is therefore needed to allow the development of new and efficient strategies [3]. Issues encountered during the development of conventional anticancer therapies can be cited, mainly caused by their poor efficacy inducing several side effects [4]. In this sense, the development of stable nanosized vehicles is of major interest to efficiently enhance the pharmacokinetics and biodistribution of the therapeutic agents. It is also important that the administered drug interacts specifically with the diseased cells, to decrease the effective therapeutic dose and preserve the healthy cells.

Controlling the drug-loaded nanocarrier particle size is crucial because it is recognised that particles with a size of about 100 nm can more efficiently target diseased cells [5], reducing recognition of the particles by the immune system. Other parameters are also important: one may cite biodegradability, biocompatibility and colloidal stability to avoid undesired phenomena (e.g., phagocytosis by macrophages, or endocytic uptake of Kupfer cells).

Micro- and nanometric capsules (also called hollow particles) have been the subject of intense research interest for many decades [6]. They are present in all fields, including agrochemistry, pharmaceuticals, cosmetics and drug delivery systems. Different encapsulation methods have been used to manufacture these hollow particles. Various approaches have been investigated in the past two decades for the synthesis of micro- and nanocarriers, such as interfacial polymerisation, phase separation from a polymer–solvent mixture [7], emulsion polymerisation, assembly of polymer-grafted amphiphilic colloids, and the layer-by-layer (LbL) method [8,9].

Microencapsulation by interfacial polymerisation was mainly developed between the end of the 1960s and the mid-1970s [10]. The process consists of the dispersion of one phase (water) containing a reactive monomer into a second immiscible phase (oil) to which a second monomer is added. Both monomers react at the droplet interface, forming a polymeric membrane. The formation of colloidal capsules via Pickering emulsions has the advantage of allowing easy modification of the capsule morphology by influencing the nature of the solvent, the quantities of the monomers and their addition rate. However, possible residues of monomers, or reagents from the polymerisation process and cross-reaction between the capsule’s drug content, might limit the potential use of these systems [11].

Liposomes are another well-known example of systems that have been used for the encapsulation of various biomaterials [12]. Although liposomes are employed as delivery systems for various species in the pharmaceutical and cosmetic fields, their limited stability in the bloodstream and low permeability for polar molecules present serious limitations for general use [13,14].

Another encapsulation method recently developed in bioimaging and targeted drug delivery is colloidal capsule synthesis with polyelectrolyte shells via the amphiphilicity-driven self-assembly of polymer-grafted nanoparticles [15]. This method offers new possibilities for the complex and controllable self-organisation of inorganic nanoparticles [16]. It consists of the adsorption of oppositely charged polyelectrolytes on the surface of colloidal particles, with the subsequent removal of the template core [8]. This simple and promising technique is used to produce micro- and nanostructured materials with controlled architecture, allowing the possibility of fabricating capsules with tuneable size, shape, shell thickness and permeability [13]. These parameters have been well investigated in many studies because of their influence on biological and pharmaceutical functions.

Polyelectrolyte multilayer micro- and nanocapsules, first introduced 20 years ago, have recently received significant interest in many areas of application, such as drug delivery, biosensing, bioimaging and catalysis [17].

Interestingly, once functionalised capsules are found inside the biological cells, controlled drug release can be initiated by various external stimuli (e.g., pH, ionic strength and temperature) [18].

Various templates (e.g., particles of calcium carbonate, gold, melamine–formaldehyde, silica, polystyrene, etc.) in the size range of tens of nanometres to 10 micrometres have been used to fabricate polyelectrolyte multilayer capsules [18]. The choice of these templates is determined by various physicochemical conditions regarding the template dissolution, the stability of the polymer shell and the tendency to aggregate.

Among the different approaches for fabricating nanovectors, the design of biopolymer-based capsules is a new expanding area of physicochemical research, involving a large range of capsule sizes, with tuneable chemical composition, size, wall thickness and mechanical properties [19]. Furthermore, these capsules have been shown to allow the encapsulation of large quantities of guest molecules [20,21]. Different strategies have been described in the literature to construct hollow nanocapsules; in this review, attention will only be paid to the LbL adsorption of biopolymers [22] onto a sacrificial porous particle, a strategy often referred to as the “templating method” [23,24,25]. The preparation of the porous particles used as the sacrificial template, the physicochemical characterisation and the applications of the polysaccharide multilayered capsules as drug carriers are reported. The large void space in the core of the capsule provides unique opportunities to load drug or bio(macro)molecules such as DNA or proteins, which can also be loaded during the LbL construction approach, allowing the incorporation of specific building bricks for targeted release. Other relevant parameters must be mentioned, such as biocompatibility, biodegradability, selective or controlled drug release, and biodistribution. Biopolymers can be used in this context as their biodegradability and biocompatibility ensure safer applications in drug delivery [26,27]. Among the biopolymers (e.g., proteins, polypeptides or biosynthetic polymers produced by living organisms), this review gives special focus to polysaccharides, which present a high number of functional groups, such as amino, hydroxyl and carboxylic acid groups, that are useful for further chemical functionalisation, to favour cell targeting or to bring stimuli-responsiveness.

In this review, recent methods of polysaccharide-based capsule preparation via LbL coatings on sacrificial porous cores are summarised by choosing journals traditionally used in the physicochemical field, such as American Chemical Society Journals, Royal Society of Chemistry, ScienceDirect, Springerlink and Wiley-Blackwell. The first section presents the preparation of the most used porous particles and their subsequent removal after coating, and the second section presents the general features of LbL assembly methods, e.g., the growth mechanism. The next section presents the most used trigger to provide drug-controlled release, while the last part presents a limited number of examples showing the use of polysaccharide-based capsules.

## 2. Polyelectrolyte Capsules Generated by Combining Porous Particles as Template and the Layer-by-Layer (LbL) Technique

The construction of hollow polyelectrolyte shells by colloid-templated consecutive polyelectrolyte adsorption followed by decomposition of the templating core is the most widespread encapsulation technique [13]. In this review, we are interested in this LbL templating approach due to its various advantages. It allows precise control of the thickness of the shell by depositing a defined number of layers on the template, which determines the capsule’s size and morphology at the same time. Numerous excellent reviews on LbL templating routes have been published, including articles by Caruso et al. [8,9] and a review by Lou et al. [28].

**Figure 1:**
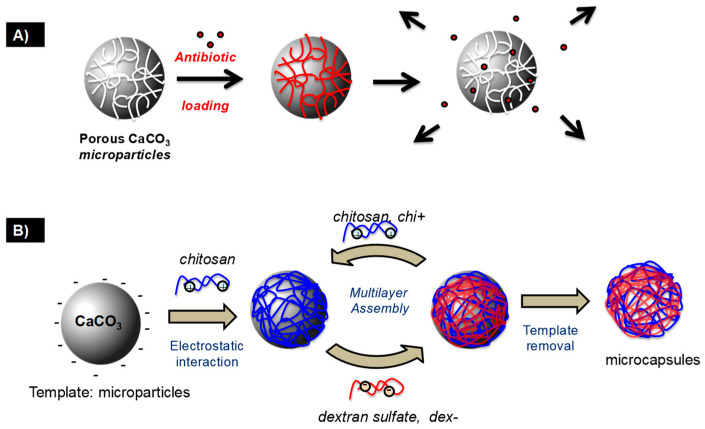
Schematic illustration of (**A**) antibiotic-loaded calcium carbonate microparticles and (**B**) the fabrication of capsules coated with polysaccharides using calcium carbonate microparticles as the template. Reprinted with permission from Ref. [29]. Copyright 2020, Elsevier.

As shown in Figure 1 [29], the mentioned process involves several steps. The most common problem is the incompatibility between the sacrificial template and the polymer shell, which may lead to complexation, i.e., self-aggregation of the polymers, and therefore to unsuccessful shell fabrication.

### 2.1. Layer-by-Layer (LbL) Assembly Based on Polysaccharides

Decher and co-workers [30,31,32] were pioneers of the multilayered polyelectrolyte concept; from this point onwards, a lot of publications have been reported based on this concept [8,33,34]. The LbL process involves an alternate dipping of substrates into aqueous polymeric solutions, including intermediate rinsing cycles for removing the polymer not strongly adsorbed onto the substrate. This is mostly required in the case of polyelectrolyte solutions because undesirable interpolyelectrolyte complexes can precipitate, thus modifying the structure and properties of the resulting material. It deserves noting that a direct LbL saturation method has also been developed [35]. This approach proposes the possibility of directly incorporating the required amount of polyelectrolytes to coat all particles on the surface, avoiding washing or purification steps. Interestingly, this method helps to avoid/limit, in the aqueous phase, the presence of free polyelectrolytes.

**Figure 2:**
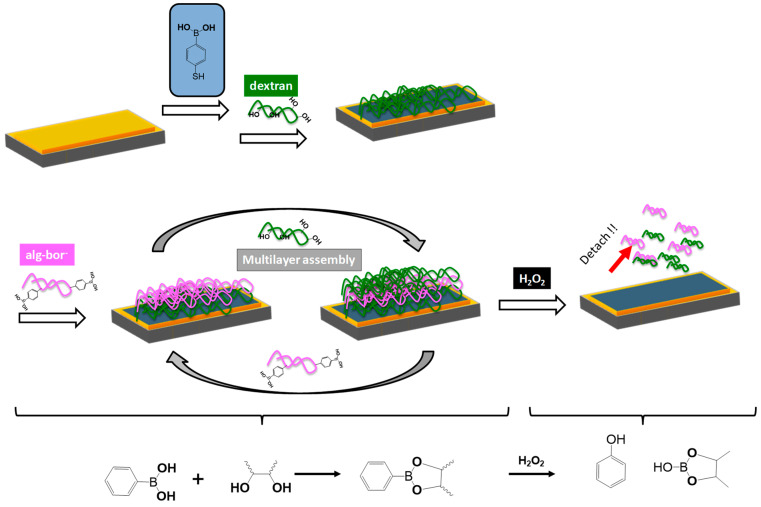
(**Top**) Representative scheme illustrating the preparation of H_2_O_2_-responsive multilayer films based on a phenylboronic acid polysaccharide derivative (alg-bor) and dextran. (**Bottom**) Illustration of the formation of a boronate ester upon the coupling of boronic acid and diol derivatives, followed by oxidation of the boronate ester to phenol by H_2_O_2._ Reprinted with permission from Ref. [36]. Copyright 2019, Elsevier.

LbL assembly driven by intermolecular interactions (e.g., electrostatic or hydrogen bonding) favours the fabrication of thin multilayer polymer films with well-controlled thickness and chemical composition, simply via the alternative deposition of judiciously selected polymers onto a substrate (Figure 2) [31].

Polymer multilayer films can grow linearly or exponentially. In the literature, the linear growth regime has been widely studied in comparison to exponential growth and is typically seen for multilayer films formed by strong polyelectrolyte pairs. The linear growth is related to the linear mass increase and thus to the film thickness due to the increase in the number of deposited polymer layers [37]. This behaviour is thought to be due to the non-diffusion of the polymer forming the multilayer film [38,39].

The advantages and limitations of employing polysaccharides vs. synthetic polymers as building blocks in multilayer films for biomedical applications are summarised in Table 1.

Polyelectrolyte pairs based on polysaccharides have been shown to present an exponential growth, which is in accordance with the fact that both mass and film thickness grow with the number of adsorbed polymers faster than linear systems. Examples include poly(L-lysine)/alginate (PLL/ALG), PLL/hyaluronic acid (PLL/HA), chitosan/ALG (CHI/ALG) and chitosan/HA (CHI/HA), to name but a few [41,42,43,44,45,46,47,48,49]. The main reason for the exponential growth has been explained as being due to the diffusion of the polycationic chains “in-and-out” of the whole film during the multilayer build-up process [50]. Salomäki and Kankare [51] reported on an acoustic-based theoretical model, which evidenced that the growth mode of the well-known CHI/HA and PLL/HA multilayers is mainly exponential. Hoda and Larson [52] explained that this kind of growth is due to the film dissolution and the existence of an energetic barrier at the multilayer film surface. It deserves to be noted that other physicochemical parameters, e.g., ionic strength, adsorption time, temperature, pH and polyelectrolyte properties (charge density, average molar mass, conformation, concentration, etc.), have been evidenced to impact multilayer assembly characteristics, such as thickness, roughness, wettability, swelling behaviour and permeability [37,47,53,54,55,56]. It should also be noted that the driving force of multilayer deposition is not limited to electrostatic forces [57], but can include other factors, such as biological affinity, covalent bonding and hydrogen bonding [58]. Hydrogen-bonded LbL assembly was first presented by Zhang and Rubner simultaneously in 1997 [59,60]. This method of assembly implies the successive deposition of polymers with either a hydrogen bond acceptor or a hydrogen bond donor [61] and is characterised by a high sensitivity to pH and ionic strength, which can be used to benefit the controlled drug release [62]. Indeed, since hydrogen-bonded LbL films mainly comprise a polyacid (carboxylic acid moiety) as the hydrogen bond acceptor, increasing the pH above its pKa causes the disruption of hydrogen bonding [61]. As a consequence, most of the hydrogen-bonded LbL films are not stable under physiological conditions. Some strategies have been developed to overcome this, such as crosslinking the resulting multilayer film [58,63,64,65] or incorporating boronic acid moieties in the polymer (e.g., alginate), which have been proven to enable the fabrication of multilayer films based on hydrogen bonding that is stable under physiological conditions [66]. For a more in-depth discussion on the LbL process, readers are kindly invited to peruse an excellent review by Borges and Mano [57], as well as the excellent review by Crouzier et al. on the interest of using polysaccharide-based polyelectrolyte multilayers [67].

The LbL approach is promising for the fabrication of multifunctional capsules. The successive deposition of polysaccharide layers is carried out on the template surface [23,25,68,69]. The selective removal of the template induces the formation of the capsules [31].

The process of preparing capsules typically involves four steps starting from the template. Step 1 consists of a surface treatment to favour the robust anchoring of the polyelectrolyte, e.g., the adsorption or grafting of (macro)molecules such as polyethyleneimine or thiol derivatives (e.g., mercaptoethane sulphonate). In the case of sacrificial templates with high charge density, step 1 may be avoided. Step 2 consists of the LbL assembly, i.e., the successive deposition of the polysaccharide pairs. Steps 3 and 4 involve the entrapment of the drug and the dissolution/removal of the porous template. These two last steps can be carried out in reverse order.

The build-up is classically investigated by QCM-D and zeta potential measurements, the resulting microcapsule morphology is analysed with CLSM, SEM and TEM, and the chemical composition with FTIR and EDS. The internal structure of the capsules is classically investigated by means of confocal and scanning electron microscopies to show the formation of capsules.

### 2.2. Porous Particle Templates

Different templates have been considered in the literature, most of which do not enable the pre-loading of the drug/component of interest. This may severely impact the application of the resulting capsules as containers for therapeutic applications because of the low encapsulation efficiencies of the cargo and the limited reproducibility associated with the diffusion process involved in the post-loading method. To overcome these drawbacks, the use of porous inorganic particles as templates, including mesoporous silica and calcium carbonate particles, has been considered. Such porous particles can be easily dissolved to form low-molecular-weight ions, limiting/avoiding osmotic stress and favouring the complete removal of the template.

Useful advantages of porous particles include their stability when dispersed in an aqueous environment when judiciously surface-modified, the possibility of entrapping either hydrophilic or hydrophobic drugs, or even both at once, a high drug-loading capacity and sustained drug release. Compared to the use of conventional polymer and/or liposome particles for the sacrificial template, the unique characteristics of inorganic porous particles allows theranostic applications to be considered [70]. Special attention has been given to porous particles of calcium carbonate (CaCO_3_) and silica in the present work, as they have been successfully used to prepare biopolymer-based capsules [66]. It should be noted that most of the weak polyelectrolyte capsules have been prepared with carbonate or silica cores.

For multilayer polymer capsule preparation, the most important step is the dissolution and total removal of the core, whose size can vary from nano- to micrometres. The template has to be inert and not affect the chemical and mechanical properties of the multilayer shell. Biopolymers are advantageous for biomedical applications; however, the design of such “biocapsules” is more challenging because of the lability and intrinsic complexity of biopolymers.

#### 2.2.1. Calcium Carbonate (CaCO_3_) Particles

Using CaCO_3_ as a sacrificial porous template is an appealing approach [71]. CaCO_3_ particles can be easily prepared from micro- to nanometre scale, and are non-toxic and low-cost [72]. The fabrication of spherical CaCO_3_ particles has been widely described in the literature, via a precipitation technique for instance [73,74], e.g., by mixing calcium chloride and sodium carbonate solutions as in Equation (1) [18].

CaCl_2_ + Na_2_CO_3_ = CaCO_3_ + 2NaCl (1)

The CaCO_3_ microparticles have a number of advantages, including facile synthesis, low cost of processing and high porosity [75], which allow the encapsulation of various biopolymers, such as proteins, enzymes or nucleic acids. CaCO_3_ particles are also characterised by their potential use as drug carriers targeting infected tissues and cells [76]. They are used to elaborate capsules that can cross physiological barriers due to their nanometric size. Nagaraja et al. [77] reported the fabrication of porous calcium carbonate nanoparticles that remained stable in aqueous solution over time. The addition of an anionic polymer, namely poly(vinylsulphonic acid) (PVSA), during the precipitation reaction allows the nucleation rate to be lowered, stabilising the surface to avoid growth or aggregation into microparticles. Biswas et al. [78] prepared oxygen-sensitive polymer nanocapsules based on PVSA-stabilised calcium carbonate nanoparticles as sacrificial templates. The authors examined the conditions for the LbL deposition of polyelectrolytes (poly(sodium 4-styrenesulphonate/poly(diallyldimethylammonium)) on CaCO_3_ colloidal templates and reported how such particles can be useful to load dyes and nanoparticles.

Campbell et al. summed up recent progress on the encapsulation of low-molecular-weight drugs into polyelectrolyte multilayer capsules templated onto CaCO_3_ particles. The drug loading/release mechanisms, benefits and limitations of such a system for biomedical applications were reported based on recent literature findings [6].

The system consists of oppositely charged natural or synthetic polyelectrolytes, which are sequentially assembled on the CaCO_3_ particles (being negatively charged) via electrostatic interactions. This is followed by a solvent-induced dissolution of the template core, using EDTA or under slightly acidic conditions. De Geest et al. developed degradable biopolyelectrolyte capsules based on this strategy, using polypeptides or polysaccharides as the enzymatically degradable components [79].

**Figure 3:**
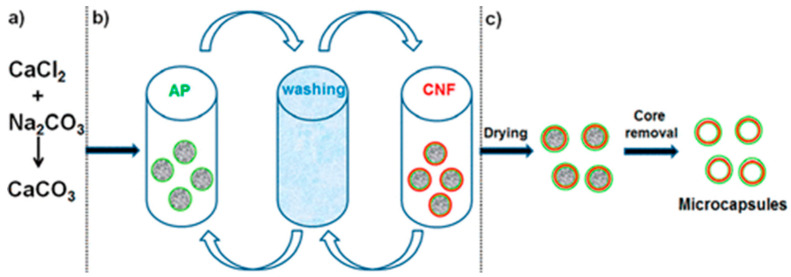
Schematic preparation of layer-by-layer (AP/CNF)_5_AP/XyG microcapsules using sacrificial CaCO_3_ microparticles: (**a**) CaCO_3_ microparticle preparation, (**b**) LbL assembly onto the surface of the CaCO_3_ microparticles, followed by (**c**) the resulting hollow microcapsules. Reprinted with permission from Ref. [86]. Copyright 2017, American Chemical Society. (AP = Pectin from apple, CNF = cellulose nanofibers, XyG = xyloglucan, LbL = layer-by-layer.)

Several researchers have reported on the templating of polysaccharide-based capsules on vaterite cores, which is one of the polymorphic forms of calcium carbonate targeted for biological purposes. For example, microcapsules composed of hyaluronic acid (HA) and poly(L-lysine) (PLL) [80]; HA and collagen (COL) [81]; poly-arginine (pARG) and heparin sulphate (HS) [82]; pARG and dextran sulphate (DS) [79]; and DS and protamine (PR) [83] have been templated upon CaCO_3_ particles. In alternate research, CaCO_3_ cores have been impregnated with a polymer matrix before the LbL coating to increase the attraction of the biopolymer layers. For instance, DS/PR and chondroitin sulphate (CS)/PR capsules have been templated on polystyrene-sulphonate-doped vaterite crystals [84,85]. We can also cite the contribution of Paulraj et al. [86], who prepared polysaccharide-based microcapsules using pectin, xyloglucan (hemicellulose) and cellulose, in the form of cellulose nanofibers (CNF), and evidenced the advantages and the loading/release of these microcapsules using calcium carbonate particles as a template (Figure 3).

**Figure 4:**
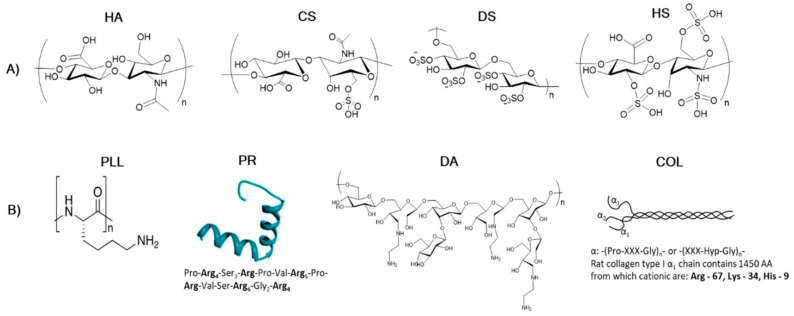
Structure of polymers utilised in the study by Campbell et al., separated into two categories: (**A**) polyanionic and (**B**) polycationic. For the proteins, the amino acid sequence (for PR) or the number of positively charged amino acids (Arg, Lys and His) per α1-helix (for collagen, COL) is given [87]. (HA = hyaluronic acid, CS = chondroitin sulphate, DS = dextran sulphate, HS = heparin sulphate, PLL = poly-L-lysine, PR = protamine, DA = dextran amine and COL = collagen.)

Previously, no systematic studies had described the mechanism of formation of multilayer biocapsules templated upon CaCO_3_ particles. Campbell et al. responded to this need by evaluating the structure–property relationship of 16 types of biopolymer capsules (Figure 4) and evidenced the correlation between the capsule formation mechanism, the shrinkage and the internal structure of their polymer composition [87].

The stability of the capsules has been correlated with the stability of the polyelectrolyte complexes formed between the biopolymers in aqueous solution without the CaCO_3_ template. They also evidenced that (i) the degree of shrinkage in the biocapsules increased with the decrease in polymer charge density and (ii) capsule adherence to the glass surface increased with the decrease in polyanion charge density, which was explained by a reduced internal compensation of charges within the multilayered shell.

**Figure 5:**
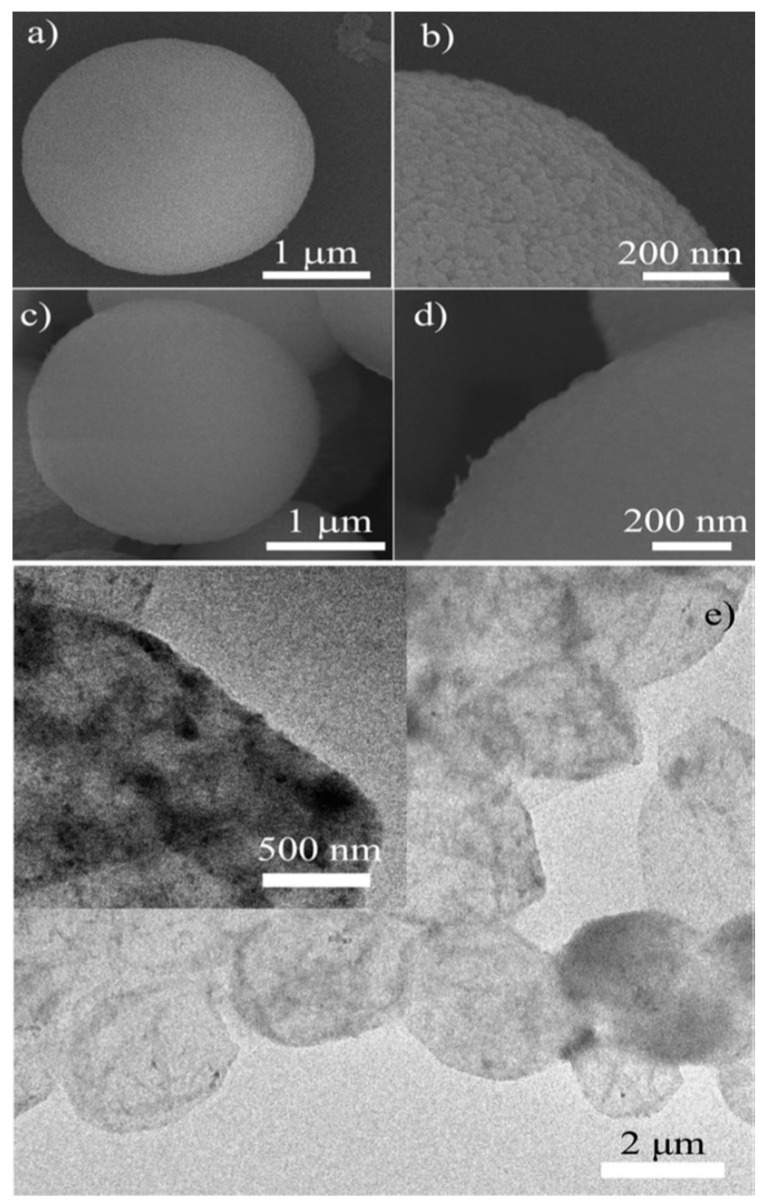
SEM images of a single porous CaCO_3_ microsphere (**a**) before and (**c**) after uploading Nile Red and assembling polysaccharide multilayers; images (**b**) and (**d**) are images with higher magnification. (**e**) Typical TEM image of Nile-Red-loaded microcapsules. The inset is a relative enlarged image of a single microcapsule. Reprinted with permission from Ref. [88]. Copyright 2012, John Wiley and Sons.

Elimination of the CaCO_3_ cores is usually induced by the addition of EDTA or using an acidic medium, which causes the dissolution of CaCO_3_ crystals and the formation of the capsules. Note that the multilayer shells may be seen to disintegrate in some cases. An example of the microscopy image of CaCO_3_ particles coated by a polysaccharide alginate dialdehyde derivative and cystamine dihydrochloride, and its subsequent removal, is shown in Figure 5 [88].

#### 2.2.2. Silica Particles

**Figure 6:**
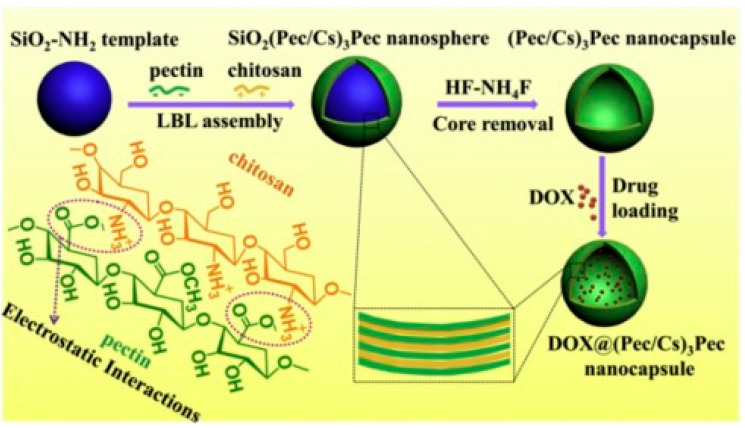
Schematic illustration of the formation process of pectin–chitosan (Pec/Cs)_3_Pec hollow nanocapsules and doxuribicin DOX@(Pec/Cs)_3_Pec nanocapsules via layer-by-layer assembly. Reprinted with permission from Ref. [95]. Copyright 2017, Elsevier. (Pec = pectin, Cs = chitosan, Dox = dorubicin.)

Since the first synthesis of mesoporous MCM-41 in 1992 [89], many approaches have been reported for the fabrication of porous silica. It has been evidenced that the templating method is the key to their preparation. By using structural templates (e.g., latex spheres, surfactant micellar systems, emulsions, colloidal crystal and even bacteria), porous solids can be obtained with highly controlled pore size distribution [90,91,92,93]. For instance, Caruso et al. [22,94] reported on the preparation of hollow inorganic silica and inorganic–hybrid spheres through the colloid template electrostatic LbL process of silica nanoparticles to form SiO_2_–polymer multilayers, followed by the removal of the templated core to obtain polyelectrolyte capsules. Ji et al. reported on pectin–chitosan nanocapsules using silica as a template. Pectin and chitosan were alternatively adsorbed on the templates via electrostatic interactions and then removed (Figure 6) [95]. Nevertheless, the size of the templates employed is currently several hundred nanometres in diameter, which can hardly form nanosized hollow particles.

Mesoporous silica particles with adjustable size (0.5–5 µm), porosity and pore size/structure have been successfully used for the entrapment of several enzymes (e.g., catalase, cytochrome C, peroxidase or even lysozyme) [96,97,98] and DNA [99]. The most limiting point when using this type of template is related to the use of hydrofluoric acid to completely remove the template, which may then limit their use for biomedical purposes [96,98,100]. Such a template is mostly employed with strong polyelectrolyte systems, but has also been extended to weak polyelectrolyte assemblies [101].

The advantages and disadvantages of the use of silica or carbonate particles as sacrificial templates to fabricate polymer capsules are presented in Table 2.

## 3. Physicochemical Triggers for Controlled Drug Release from Polysaccharide-Based Capsules for Biological Application

Capsules have intrinsic characteristics such as low-density refractive indices, high surface-to-volume ratio and also thermal expansion coefficients. Furthermore, through the judicious choice of the polyelectrolyte used during the LbL process, additional properties can be provided, such as biodegradability, biocompatibility and stimuli-responsiveness. This finding accounts for the large interest in using these capsules for biomedical imaging and drug delivery, where their capacity for entrapping sensitive cargoes (e.g., therapeutics, fluorescent markers, field-responsive agents, etc.) has been widely investigated. Moreover, polyelectrolyte capsules can be employed to gain information on specific biological processes according to the particle characteristics, such as size, shape, composition and surface functionality [102]. Designing biocapsules containing a multicompartment structure is a powerful way to enhance their activity for biological purposes by bringing multiple new functionalities, which can be useful for developing systems for both therapy and diagnosis.

Biodegradable and biocompatible capsules, including the polysaccharide capsules discussed herein, could be useful for multiples reasons: (1) the hollow core, with a large inner space, allows the encapsulation of a large amount of drug and may also protect it from degradation; (2) the possibility of preparing small capsules renders them compatible with different methods of administration (e.g., intravenous injection) and leads to a rapid absorption and drug release behaviour; (3) the biocompatibility and biodegradability of the polysaccharides renders them less toxic to cells. Polysaccharides have been widely used in biomedical applications due to the above cited advantages and their low cost. It deserves noting that even if polysaccharide-based capsules are widely used in current biomedicine, there are only few reviews on related design considerations. For instance, De Geest et al. showed polyelectrolyte microcapsules for in vitro and in vivo drug delivery and their use as biosensors [103].

In the following section, judiciously selected examples from the literature will be reported, i.e., examples of the major interest or benefits of using polysaccharide-based capsules for drug delivery.

Drug encapsulation is generally considered to achieve the controlled release of active molecules. A particularly effective strategy is the use of triggers to induce release, i.e., upon a specific stimulus, at the desired place and time of action. It is now recognised that the use of intelligent delivery systems that undergo morphological changes in response to varying physiological environments can improve delivery efficiency in terms of release kinetics, dose and localisation. In this review, we will briefly consider the most commonly used triggers, which rely on the use of heat, light, redox effects and pH to achieve active release. Note that drug release has also been successfully triggered by other mechanisms, such as enzymes [104], mechanical shear [105] or ultrasound activation [106].

### 3.1. Temperature

Heat-sensitive drug delivery systems are among the most studied, mainly because tumour tissues have abnormal temperature gradients compared to normal tissues and are very sensitive to temperature increases [107,108,109,110]. In this sense, Zhang et al. prepared capsules composed of Pluronic^®^ F127 and chitosan for the specific intracellular release of low-molecular-weight compounds, namely ethidium bromide [111]. The thermal responsiveness of the polysaccharide-based capsules was exploited. For instance, the particle size varied from 37 nm at 37 °C to 240 nm at 4 °C. This was accompanied by a permeability change. At low temperatures, the permeability of the capsule wall was enhanced, enabling free diffusion of the encapsulated cargoes. Intracellular delivery was performed in cytosol of both cancerous and non-cancerous mammalian cells. In another study, the same research group investigated the controlled intracellular release of trehalose (glucose disaccharide), which is a highly hydrophilic molecule [112]. A major result of this work was the low cytotoxicity of the bio-based capsules.

### 3.2. pH

**Figure 7:**
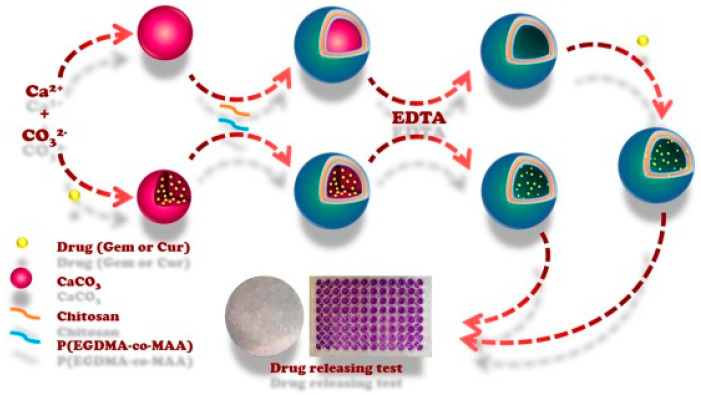
Schematic representation of pH-sensitive capsule fabrication and drug delivery system. Reprinted with permission from Ref. [120]. Copyright 2022, Elsevier. (EDTA = ethylenediaminetetraacetic acid, P(EGDA-co-MAA) = poly(ethylene glycol dimethacrylate-co-methacrylic acid), Gem = gemcitabine, Cur = curcumin.)

Using pH as a trigger suggests that changes in a materials’ properties/functionality can be induced by varying the concentration of hydronium ions. The most obvious change observed in nature is the pH variation from the acidic medium in the stomach to the more neutral pH of the intestines (stomach (pH 1–2); intestine (pH 8.4); or endosome (pH 6.0–6.5)). Decreases from the physiological pH value of 7.4 to lower pH values can be seen in the extracellular media of tumour tissue (pH 6.6–7.4) and endosomes (pH = 5). pH-triggered particles are designed with the aim of inducing a change in hydrophilicity/hydrophobicity upon protonation/deprotonation equilibrium in the particle. Previously reported pH–responsive systems include esters [113], acetals [114], boronic acids [115], hydrazones [116], imines [117] and functional groups. To ensure drug release, the cleavage of acid/base-sensitive bonds must induce a significant change in permeability, which is often achieved via structural fragmentation, depolymerisation or polarity change. In contrast, polyelectrolyte-based carriers are responsive to pH variations and the corresponding variations in ionisation state through protonation or deprotonation changes, electrostatic interaction forces and the overall carrier hydrophilicity. A combination of both effects was reported by Almutairi and co-workers, who fabricated nanoparticles from a copolymer of ketal and aminoester monomers [118]. The final particles presented a burst release profile at lower pH, while protonation implied a swelling effect, leading to an enhancement of the ketal cleavage throughout the nanoparticles. pH-responsive drug delivery systems are also very promising for intracellular drug delivery purposes due to the fact that the pH falls between intracellular endosomes, lysosomes and physiological media. For example, Zhao et al. nicely described the fabrication of biodegradable drug delivery multilayer polyelectrolyte capsules composed of chondroitin sulphate/poly(L-lysine) presenting pH-tuneable loading and release properties [119]. Bovine serum albumin tagged by fluorescein isothiocyanate was entrapped as a model drug. The authors demonstrated that the loading increased at a pH value near the isoelectric point of bovine serum albumin (pH 5.0) and also released faster at higher pH. Kazemi-Andalib et al. reported on the preparation of bio-based capsules using a simple and facile procedure that was not labour-consuming, i.e., the fabrication of microcapsules through the sequential deposition of chitosan and poly(ethylene glycol dimethacrylate-co-methacrylic acid) layers (Figure 7) [120].

**Figure 8:**
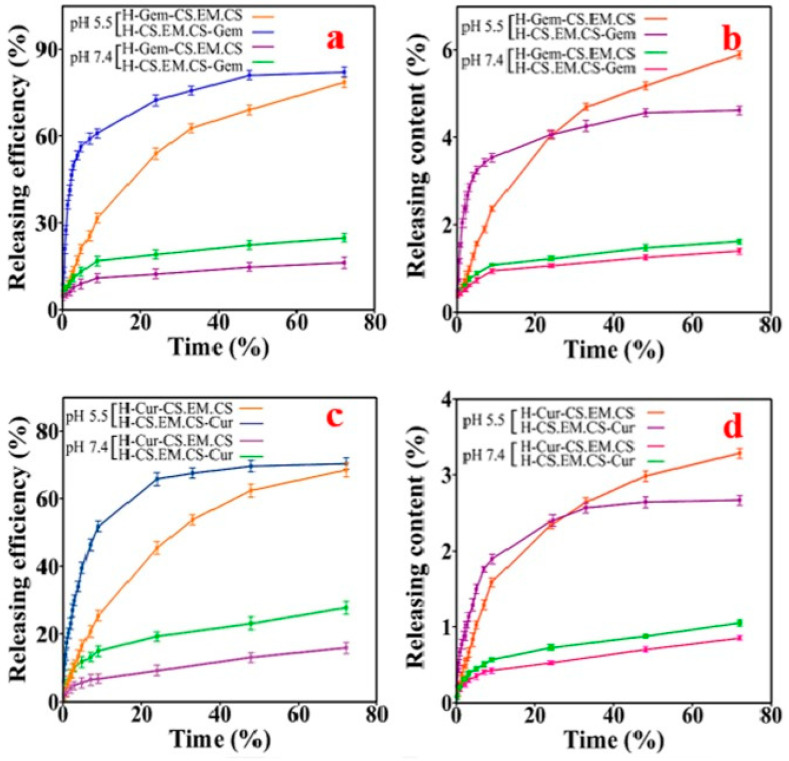
The release profile of drugs from microcapsules: Gem (**a**) RE%, (**b**) RC% and Cur (**c**) RE%, (**d**) RC%. Reprinted with permission from Ref. [120]. Copyright 2022, Elsevier. Gem = gemcitabine, Cur = curcumin, RE = releasing efficiency, RC = releasing content, H = hollow microcapsule, CS = chitosan, EM = poly(ethylene glycol dimethacrylate-co-methacrylic acid.)

They evidenced that the as-prepared polysaccharide-based capsules were pH-sensitive and could be used in cancer therapy. They encapsulated two drugs, namely gemcitabine and curcumin, and then studied the drug sustained-release behaviour in different release media, including a buffer solution at pH 5.5 to simulate the tumoural acidic environment and at pH 7.4 for physiological conditions (Figure 8) [120].

### 3.3. Redox-Triggered Chemistry

**Figure 9:**
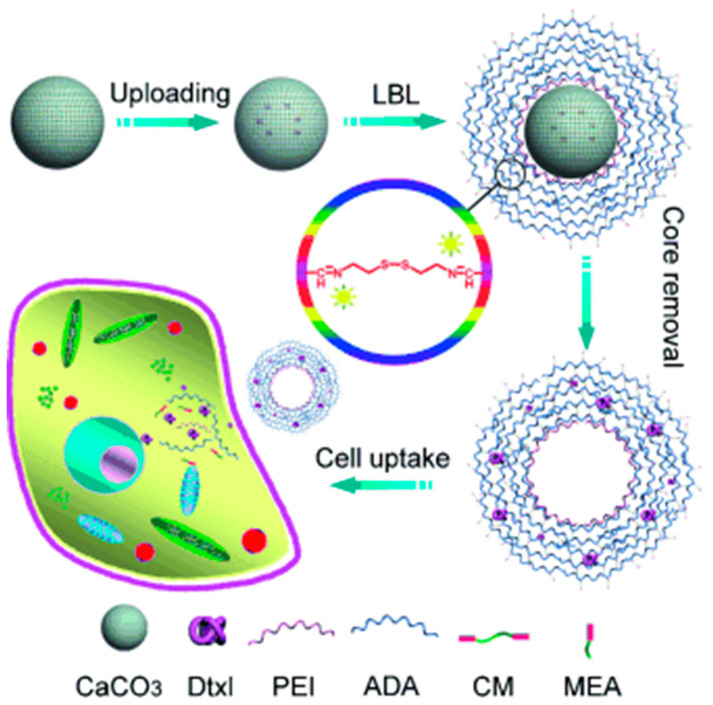
LBL assembly of autofluorescent polysaccharide-based microcapsules uploading docetaxel (Dtxl) against tumour cell proliferation. The formulated Schiff base and disulphide bonds form capsules with pH- and redox-responsive properties for pinpointed intracellular delivery based on physiological differences between the intracellular and extracellular environment. The as-obtained microcapsules could be degraded into ADA and mercaptoethylamine (MEA). Reprinted with permission from Ref. [88]. Copyright 2012, John Wiley and Sons. (PEI = poly(ethyleneimine), ADA = alginate dialdehyde, CM = cystamine dihydrochloride.)

Thiol–disulphide (SH—S-S) chemistry is of particular interest for biomedical purposes as both are exchangeable and reversible [121]. Indeed, a series of low-molecular-weight thiols have been evidenced to be key in redox-mediated processes in cells, the most important being glutathione (GSH), a tripeptide composed of glutamic acid, cysteine and glycine. It is recognised that a significant difference in redox potential exists between the intracellular and extracellular compartments. This change is linked to the higher GSH concentrations in cytosol than in the extracellular media [122]. Interestingly, cells presenting a high proliferation rate, as in the case of cancer cells, contain a high level of GSH specifically in the nucleus [123]. In the cellular environment, GSH behaves as a reducing agent and is able to disrupt carriers containing the S-S linkage, resulting in the release of drugs [124]. Biopolymer capsules that can be subjected to reduction by GSH are thus promising and attractive for specific intracellular drug delivery [125,126]. For instance, Gao et al. prepared capsules containing a modified polysaccharide—negatively charged alginate dialdehyde—and a small biomolecular cross-linker—positively charged cystamine dihydrochloride (containing a disulphide linkage) [88,120]. The preparation process for uploading hydrophobic anticancer drugs against tumour cell proliferation is shown in Figure 9.

**Figure 10:**
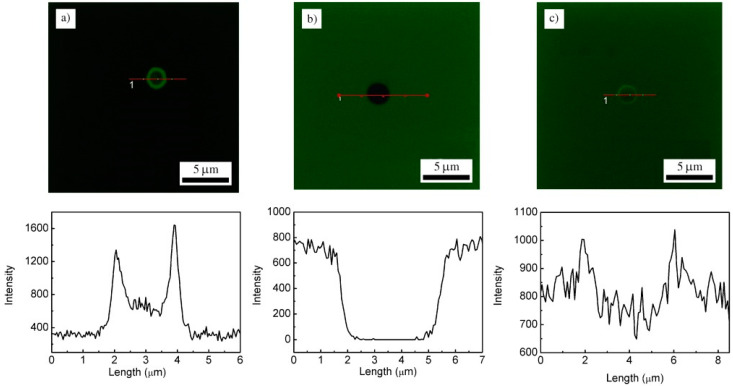
CLSM images and the relative fluorescence intensity profiles of (ADA/CM)5 microcapsules in different media with FITC-dextran (2000 KDa) as a reporting molecule: (**a**) pH 5.0, without DTT; (**b**) pH 7.4, without DTT; (**c**) pH 7.4, with 10 mm DTT. Reprinted with permission from Ref. [88]. Copyright 2012, John Wiley and Sons. (CLSM = confocal laser scanning microscope, ADA = alginate dialdehyde, CM = cystamine dihydrochloride, DTT = dithiothreitol.)

Gao et al. [88] then demonstrated the redox-responsive properties of microcapsules by investigating their permeability in different redox solutions using FITC-dextran (2000 KDa) as a molecular probe, as shown in Figure 10. They evidenced that the addition of a reducing agent (DTT: dithiotreitol) induced an increase in the permeability of the multilayer capsule, which was related to the cleavage of the disulphide linkage within the formed multilayers. This strategy was then proven to allow the intracellular delivery of a cancer drug (docetaxel).

### 3.4. Glucose-Triggered Chemistry

**Figure 11:**
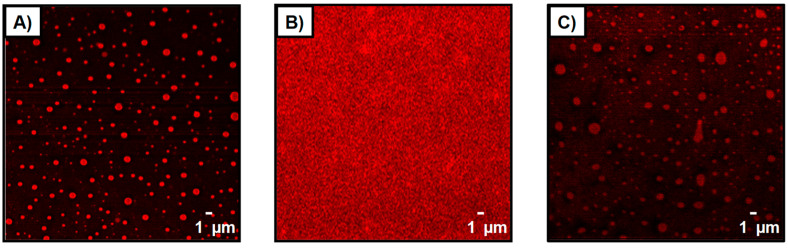
Confocal microscopy image of microcapsules consisting of (alg-bor/PVPON)_5_ loaded with rhodamine derivative (**A**) before and (**B**) after the addition of a solution containing glucose at pH = 8 and (**C**) after the addition of a solution containing glucose at pH = 2 (630× magnification). Reprinted with permission from Ref. [66]. Copyright 2019, Elsevier. (PVPON = polyvinylpyrrolidone, alg-bor = alginate derivative modified with phenylboronic acid.)

**Figure 12:**
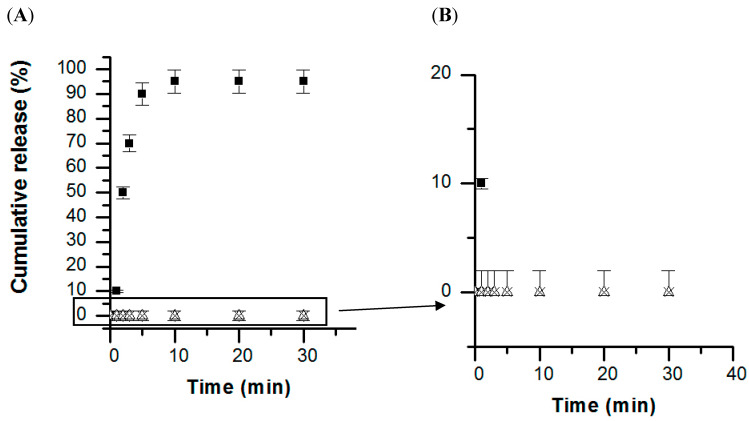
(**A**) Release of insulin from (alg-bor/PVPON)_5_ capsules pH 8 in the presence of glucose solution (1 g·L^−1^) at Δ pH 2, ■ pH 8 and x pH 8 without glucose; (**B**) a zoom-in of graph A. Reprinted with permission from Ref. [66]. Copyright 2019, Elsevier. (PVPON = polyvinylpyrrolidone, alg-bor = alginate derivative modified with phenylboronic acid.)

Developing glucose-sensitive drug delivery systems is another interesting system, as glucose has a crucial biological function in living systems [127]. Using phenylboronic acid is known to allow the development of a glucose-responsive carrier, as the chemical function of the phenylboronic acid allows the formation of covalent bonds with diol derivatives, such as polysaccharides, under specific physicochemical conditions [128]. More precisely, the complexation is only possible in a pH range that is equal to the pKa of the used boronic derivative (which is generally pKa~9) [127,128,129], where phenylboronic vs. phenylboronate are in equilibrium. Complexation with glucose and boronic acid allows the equilibrium to shift in the direction that enhances the charged phenylborates because of the formation of charged borates, which can form a stable complex with glucose in aqueous solution. Most of the developed multilayer capsules are based on the total destabilisation of the film upon contact with a sugar solution up to a critical concentration [128,130]. For instance, Levy et al. reported on the fabrication of glucose-sensitive polysaccharide-based capsules, stabilised via ester bonds formed between mannan and phenylboronic acid moieties grafted onto poly(acrylic acid) [130]. The competitive binding of the saccharides and mannan with the boronic acid function inside the multilayer assembly resulted in the destabilisation of the film. A complementary route to the sugar-induced total dissolution of polyelectrolyte multilayers has recently been reported by Belbekhouche et al. [72], where glucose-sensitive polyelectrolyte microcapsules based on (alginate/chitosan) were reported, and the glucose response was linked to a variation in the swelling behaviour of the multilayer film. Capsules based on polyvinylpyrrolidone (PVPON) and an alginate derivative modified with phenylboronic acid (alg-bor) moieties have also been reported in the aim of designing glucose-sensitive capsules [66]. The glucose-sensitive character was related to the concentration of glucose inducing a disassembly/destructuring of the multilayer film. This behaviour was used for a specific fluorescent cargo release triggered by glucose (Figure 11) and was extended to a specific insulin release (Figure 12).

## 4. Conclusions, Current Challenges and Future Directions

This review reports on the interest in developing polysaccharide-based capsules for biological purposes. Since multilayer polysaccharide films are relatively nontoxic and biodegradable, and may present specific bioactivities (e.g., anti-inflammatory), there is a high demand for the design of polysaccharide-based capsules. We have focused on the templating method to prepare such particles using porous particles (silica and carbonate particles). Mesoporous carbonate particles have become the most popular sacrificial templates for such biopolymer deposition because of their nontoxicity, biocompatibility and highly implemented internal structure, which can be used as an ideal network for drug loading, as well as being scalable and low-cost. Polysaccharide-based capsules with varied chemical compositions, size, mechanical properties and stimuli-responsiveness can be successfully designed with the aim of developing controlled drug release systems in response to physical–chemical triggers. As classical capsule wall polymers, widely available low-cost polysaccharides such as alginate and chitosan are the primary choice for researchers. Polysaccharide-based capsules with multilayer shells were reported to be fabricated herein by a method combining porous particles as template and the LBL process required several steps which tend to increase the preparation cost [131]. These several preparation steps induce low yield and a high cost of the overall strategy. These are obstacles to the promotion of capsules prepared via this procedure in the market. Similar to oral capsules already on the market, the design approach of polysaccharide-based capsules gradually tends to be convenient and simple to prepare. This needs researchers to continuously implement and explore more convenient methods and approaches for the elaboration of polysaccharide-based capsules.

Using polysaccharide capsules as vectors relies on an efficient encapsulation of the bioactive, the preservation of its biological activity and good physicochemical properties of the capsules (size, flexibility, capsule stability, etc.). These are the main critical factors that should be taken into account when designing these systems for therapeutic purposes. Considering the complexity of biological entities in terms of temperature-, ionic strength- and pH-dependence, the development of a universal protocol to load bioactive compounds into biopolymer capsules still remains a challenging task. Similar to the oral capsules already found on the market, the design approach of polysaccharide-based capsules gradually tends to become easier and more straightforward to prepare. This requires researchers to continue developing and exploring more convenient approaches for the fabrication of polysaccharide-based capsules. In addition, capsules for in vivo applications are still challenging.

For biological purposes, the key challenge remains the need to implement a synthetic carrier that mimics biomimetic approaches, to favour the translation of robust systems. Even though considerable progress has been made in the fabrication of biopolymer capsules behaving as micro-/nanoreactors, they still lack the complexity of biological cells, in which multiple biochemical reactions are simultaneously carried out in response to the microenvironment. To overcome this issue, the development of multicompartment particles are being extensively explored via polymersomes-in-polymersomes, liposomes-in-liposomes, polymersomes-in-liposomes, liposomes-in-polymersomes, capsules-in-capsules and polymersomes-in-capsules [132]. Biological barriers and multidrug resistance are critical targets for several drug delivery carriers. In the future, the discovery of new polysaccharide-based materials is expected to allow broader strategies for capsule design and biomedical use. Therefore, to enhance the clinical translation of biopolymer capsules as therapeutic reactors, significant efforts are needed in the future to validate the performance of designed capsules in biological systems [133,134].

## Figures and Tables

**Table 1:** Synthetic polyelectrolytes vs. polysaccharides for multilayer build-up for biomedical purpose.

	Synthetic Polyelectrolytes [40]	Polysaccharides [40]
Benefits	- low polymolecularity index and highly controlled quality - large choice of charge densities and chemical structure - large working range of ionic strength and pH - easy chemical modification	- most biodegradable and biocompatible - biomimetic - intrinsic biological properties: present in the pericellular coat, interaction with bioactive compounds, etc. - easy chemical modification - large choice of charge densities
Limitations	- most non-biodegradable - no specific bioactivity - toxicity when degraded	- often polymolecular and non-pure at 100% - aqueous solubility issues (limiting ionic strength and pH working range) - some chemical modification can be difficult (poor solubility in solvent, poor reactivity of the group, low charge density)

**Table 2:** Pros and cons of using porous silica and calcium carbonate particles as templates for polyelectrolyte capsule preparation for biological purposes.

Sacrificial Template	Pros	Cons
Calcium carbonate particle [6,72,73,74,75]	- biocompatible/non-toxic - low cost - high loading capacity (high surface area (1500 m^2^/g)) - possible to preload an active component inside the particle’s porosity - mild conditions to remove the template (EDTA solution or slightly acidic conditions) - complete template removal	- difficult to obtain nanosized particles that are colloidally stable in aqueous solution - poor control over the particle size and polydispersity index
Mesoporous silica particle [22,94,98,100]	- biocompatible - commercially available in a large range of sizes (50 nm to 15 µm) - high loading capacity (high surface area (1500 m^2^/g)) - possible to preload an active component inside the particle’s porosity - complete template removal	- requires hydrofluoric acid to remove the template

## Data Availability

Not applicable.
